# Testing speech biomarkers against cognitive and neural signatures of Alzheimer's dementia

**DOI:** 10.1002/alz70856_107220

**Published:** 2026-01-08

**Authors:** Ivan Caro, Gonzalo Nicolás Pérez, Joaquín Valdez Bisé, Joaquín Ponferrada, Franco Javier Ferrante, Alejandro Sosa Welford, Lara Gauder, Luciana Ferrer, Agustin Ibanez, Andrea Slachevsky, Adolfo M Garcia

**Affiliations:** ^1^ Consejo Nacional de Investigaciones Científicas y Técnicas (CONICET), Buenos Aires, Argentina; ^2^ Cognitive Neuroscience Center, University of San Andrés, Victoria, Buenos Aires, Argentina; ^3^ Universidad de Buenos Aires, Buenos Aires, Argentina; ^4^ Department of Psychiatry, School of Medicine, Pontificia Universidad Católica de Chile, Santiago, Chile; ^5^ University of Buenos Aires, Ciudad Autonoma de Buenos Aires, CABA, Argentina; ^6^ University de Buenos Aires, Ciudad autonoma de buenos aires, CABA, Argentina; ^7^ Global Brain Health Institute (GBHI), University of California San Francisco (UCSF); & Trinity College Dublin, Dublin, Ireland; ^8^ Latin American Brain Health Institute (BrainLat), Universidad Adolfo Ibañez, Santiago, Chile; ^9^ Memory and Neuropsychiatric Center (CMYN) Neurology Department, Hospital del Salvador and Faculty of Medicine, University of Chile, Santiago, Region Metropolitana, Chile; ^10^ Neuropsychology and Clinical Neuroscience Laboratory (LANNEC), Physiopathology Department ‐ ICBM, Neuroscience and East Neuroscience Departments, Faculty of Medicine, University of Chile, Santiago, Chile; ^11^ Geroscience Center for Brain Health and Metabolism (GERO), Santiago, Chile; ^12^ Servicio de Neurología, Departamento de Medicina, Clínica Alemana‐Universidad del Desarrollo, Santiago, Chile; ^13^ Departamento de Lingüística y Literatura, Facultad de Humanidades, Universidad de Santiago de Chile, Santiago, Chile

## Abstract

**Background:**

Automated analysis of word properties (WP) and speech timing (ST) offers a cutting‐edge digital method for identifying scalable markers of Alzheimer's disease dementia (ADD). Quantification of lexical features during speech can not only detect ADD cases but also predict cognitive performance and associated anatomical‐functional brain patterns. However, the clinical value of WP/ST markers remains uncertain, as no study has yet compared their discriminatory capacity to standard cognitive and neural measures, casting doubt on their utility for under‐resourced settings that lack such conventional tools.

**Method:**

We recruited 33 ADD patients and 33 healthy controls from a carefully characterized cohort. Each participant completed two 1‐minute verbal fluency tests, cognitive evaluations (addressing general cognitive skills, attention, set‐shifting, and working memory), and MRI scans. Separate machine learning classifiers were trained using (i) WP/ST features from the fluency tasks (obtained through the Toolkit to Examine Lifelike Language [TELL] app), (ii) scores from the cognitive tests, and (iii) volumetric features from the imaging protocol. The best‐performing model for each feature set was then evaluated based on mean AUC, accuracy, and 95% confidence intervals. Additionally, we identified the top discriminatory WP/ST markers through feature importance analysis and explored their correlation with gray matter volume.

**Result:**

Classification outcomes revealed comparable performance between WP/ST features (AUC = 0.88; accuracy = 0.81) and both cognitive (AUC = 0.86; accuracy = 0.79) and imaging (AUC = 0.92; accuracy = 0.88) features, with overlapping confidence intervals suggesting similar discriminability. The top five WP/ST features were word concreteness, semantic variability and frequency for the semantic fluency task, and semantic variability and granularity for the phonemic task. Across all participants, word frequency showed a negative correlation with the volume of right prefrontal areas involved in executive processing.

**Conclusion:**

**Using only two minutes of audio recordings, our digital speech analysis pipeline can identify ADD with performance comparable to gold‐standard measures, driven by features linked to brain regions implicated in cognitive symptoms typical of ADD. Overall, WP/ST analyses seem non‐inferior to standard diagnostic measures, highlighting their potential as a scalable and low‐cost tool for dementia assessment**.

